# Modeling Adaptive Regulatory T-Cell Dynamics during Early HIV Infection

**DOI:** 10.1371/journal.pone.0033924

**Published:** 2012-04-19

**Authors:** Michael Simonov, Renata A. Rawlings, Nick Comment, Scott E. Reed, Xiaoyu Shi, Patrick W. Nelson

**Affiliations:** 1 Department of Mathematics, University of Michigan, Ann Arbor, Michigan, United States of America; 2 Center for Computational Medicine and Bioinformatics, University of Michigan, Ann Arbor, Michigan, United States of America; 3 Department of Biomedical Engineering, University of Michigan, Ann Arbor, Michigan, United States of America; Dana-Farber Cancer Institute, United States of America

## Abstract

Regulatory T-cells (Tregs) are a subset of CD4^+^ T-cells that have been found to suppress the immune response. During HIV viral infection, Treg activity has been observed to have both beneficial and deleterious effects on patient recovery; however, the extent to which this is regulated is poorly understood. We hypothesize that this dichotomy in behavior is attributed to Treg dynamics changing over the course of infection through the proliferation of an ‘adaptive’ Treg population which targets HIV-specific immune responses. To investigate the role Tregs play in HIV infection, a delay differatial equation model was constructed to examine (1) the possible existence of two distinct Treg populations, normal (nTregs) and adaptive (aTregs), and (2) their respective effects in limiting viral load. Sensitivity analysis was performed to test parameter regimes that show the proportionality of viral load with adaptive regulatory populations and also gave insight into the importance of downregulation of CD4^+^ cells by normal Tregs on viral loads. Through the inclusion of Treg populations in the model, a diverse array of viral dynamics was found. Specifically, oscillatory and steady state behaviors were both witnessed and it was seen that the model provided a more accurate depiction of the effector cell population as compared with previous models. Through further studies of adaptive and normal Tregs, improved treatments for HIV can be constructed for patients and the viral mechanisms of infection can be further elucidated.

## Introduction

Although the dynamics of CD4^+^ and CD8^+^ cells have been well characterized, in HIV infection, there is currently a lack of understanding concerning the role of regulatory T-cells, or Tregs in viral dynamics [1]. Tregs are a class of CD4^+^ cells which limit the activation and expansion of immune cells, including autoreactive CD4^+^ cells and CD8^+^ cells. Initial studies provided evidence that the Treg response to HIV was beneficial, limiting immune exhaustion and immune-mediated tissue damage [2]–[3]. Conversely, Tregs have been observed to contribute to the onset of immune dysfunction and to prevent a successful immune response [3]–[5]. Finally, there is evidence that the role of Tregs throughout infection may follow a more dynamic behavior, changing its behavior at different stages of infection [3] [6]. Divergent reports on Treg activity can be attributed in part to experimental obstacles involved in studying their dynamics *in vivo*. Patient data for Tregs are largely non-existent due to the absence of accurate surface markers to characterize the population. Without selective markers, computational modeling is paramount in providing insight into T-cell regulation during HIV infection, specifically examining adaptive Treg behavior.

To further exacerbate the experimental problem in HIV infection, there is evidence that multiple subsets of Tregs exist: normal Tregs, as well as a HIV-specific adaptive Tregs [3] [6]–[8]. Normal Tregs (nTreg), the body’s naturally occuring regulatory T-cells, are present in the early stages of infection, but through some unknown mechanism, HIV may adapt the activity of nTregs, to the benefit of the virus; this modified class of Tregs is deemed adaptive Tregs (aTreg) [6].

Adaptive Tregs are believed to be deleterious for the patient by contributing to viral proliferation and poor immune activity. Although there are no known markers for adaptive Tregs, their existence may have a distinct effect on HIV dynamics [6]. In our model, we included both nTregs and aTregs, matching simulations to patient data and constructed an understanding of whether the two subsets could biologically exist together with differing effects. Our model focuses on the effect Tregs have on sharply declining viral load during acute infection and the examination of the importance of CD8^+^ activation and CD4^+^ population limitation as well as its subsequent steady state levels during latency.

## Results

### Model Provides Realistic Effector Cell Dynamics which are Lacking in Previous Models

**Figure 1:**
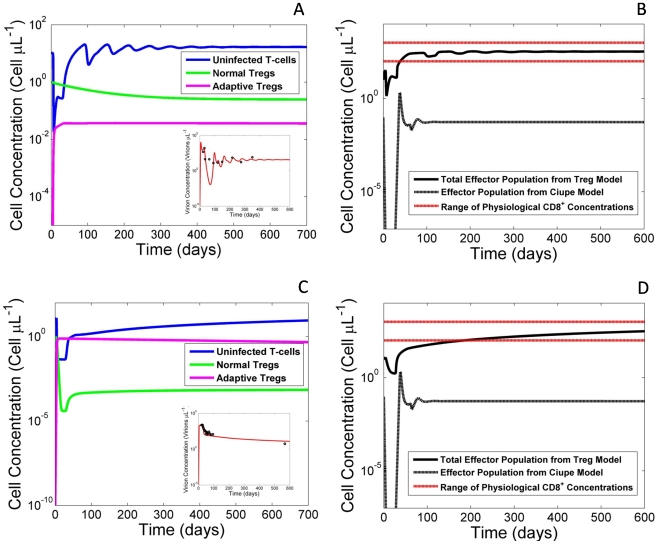
Treg model shows improved effector cell dynamics over previously published fits. (A,C) Plot of modeled uninfected T-cell population (blue), normal Treg (green) and adaptive Treg populations (purple). Models correspond to best fits to the viral load data from two separate representative patients. (inset) Patient data is shown (circular data points), and viral load is shown (red). Parameter values for simulations can be found in Table 1. (B,D) Comparative plots of effector populations using Treg model as compared to a previously published model [9] as well as physiological ranges for effector cells [10] as seen by the red horizontal lines in B and D. In all cases, we notice that our model does not dip to unrealistic ranges initially as does the Ciupe model. However, the viral load fits between both models are nearly the same suggesting a degree of robustness between both models.

Through data fitting, the model was initially tested to ensure normal viral behavior could be replicated. Consistent with previous fits, two main behaviors were observed - oscillatory as well as steady state dynamics [9] (Fig. 1A, C). The model was capable of reproducing the acute viral infection and proceeding either to steady state or damped oscillations. Comparing our fits to a previously published study (Cuipe et. al), lacking normal and adaptive regulatory T-cells, reveals that similar dynamics could be obtained through the inclusion of a regulatory T-cell population. Notably however, Treg fits showed improvements in simulating effector cell populations, within the first few weeks of infection, compared to previous models (Fig. 1B, D). In comparing fits between the Ciupe model and our model, we find comparable fits; however, when we examine the ranges of effector cells, we find a significant improvement in our model. Our model is noted as staying within physiologically relevant effector population ranges for the majority of the simulation, whereas the Ciupe model never enters physiological ranges. In fact, the Ciupe model displays a sharp decline in effector populations early in simulation not seen biologically. Comparing the effector populations with data on effector populations reveals that our model provides a more biologically relevant dynamic with comparable fits to viral load [10]. We believe these improvements are due to the usage of dual equations modeling the effector cell population. Through compartmentalization of effector cells into immature and mature populations, we were able to avoid sharp drops in these cells during acute infection and construct a method of modeling effector populations within realistic physiological bounds.

**Table 1:** Treg model parameter ranges and descriptions.

Parameter	Dimensions	Description	Patient 2 Fit	Lower	Upper	Patient 8 Fit	Lower	Upper
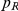	cells/( 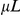 *day)	Production of nTregs	0.0023	0	0.070	3.519E-05	0	1.070
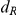	1/day	Death rate of nTregs	0.0093	[9]	[9]	0.0458	[9]	[9]
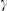	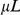 /(virus*day)	Probability of nTreg 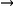 aTreg transition	6.621E-7	6.677E-08	2.44E-4	3.857E-4	9.472E-07	0.0313
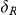	1/day	Death rate of aTreg	2.218E-4	[9]	[9]	7.287E-4	[9]	[9]
N	virus/cell	Burst size of infected T-cells	2205	[9]	[9]	6716.452	[9]	[9]
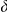	1/day	Death rate of Infected T-cells	1.094	[9]	[9]	0.06	[9]	[9]
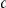	1/day	Clearance rate of virus	0.6167	[9]	[9]	2.552	[9]	[9]
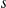	cells/( 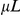 *day)	Production of T-cells	0.7672	[9]	[9]	0.855	[9]	[9]
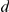	1/day	Death of T-cells	8.214E-4	[9]	[9]	0.00122	[9]	[9]
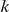	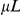 /(virus*day)	Probability of interaction of T-cell with virus resulting in infection	9.964E-4	[9]	[9]	0.0087	[9]	[9]
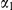	1/day	Downregulation rate of nTregs on T-cells	0.601	0.0653	5.464	0.00218	0	1.627
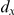	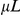 /(cell*day)	Death of infected T-cells by mature effectors	0.3755	[9]	[9]	0.0671	[9]	[9]
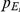	cells/( 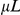 *day)	Production of immature effectors	0.545	0.0082	3.898	0.525	0.247	1.047
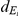	1/day	Death rate of immature effectors	0.0204	[9]	[9]	1.88E-4	[9]	[9]
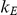	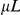 /(virus*day)	Probability of interaction between virus and immature effector resulting in mature effector	3.699E-4	0	9.249E-05	1.411E-05	0	5.312E-05
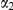	1/day	Downregulation rate of nTregs on immature effectors	0.0485	0	0.111	0.0020	0	15.164
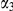	1/day	Downregulation rate of aTregs on mature effectors	0.403	0	254.17	1.211E-08	0	0.0132
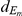	1/day	Death rate of mature effectors	0.0124	[9]	[9]	0.00121	[9]	[9]
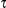	day	Time delay of emergence of mature effector population	26.2546	[9]	[9]	23.4321	[9]	[9]

### Sensitivity of Normal Tregs on Viral Load

**Figure 2:**
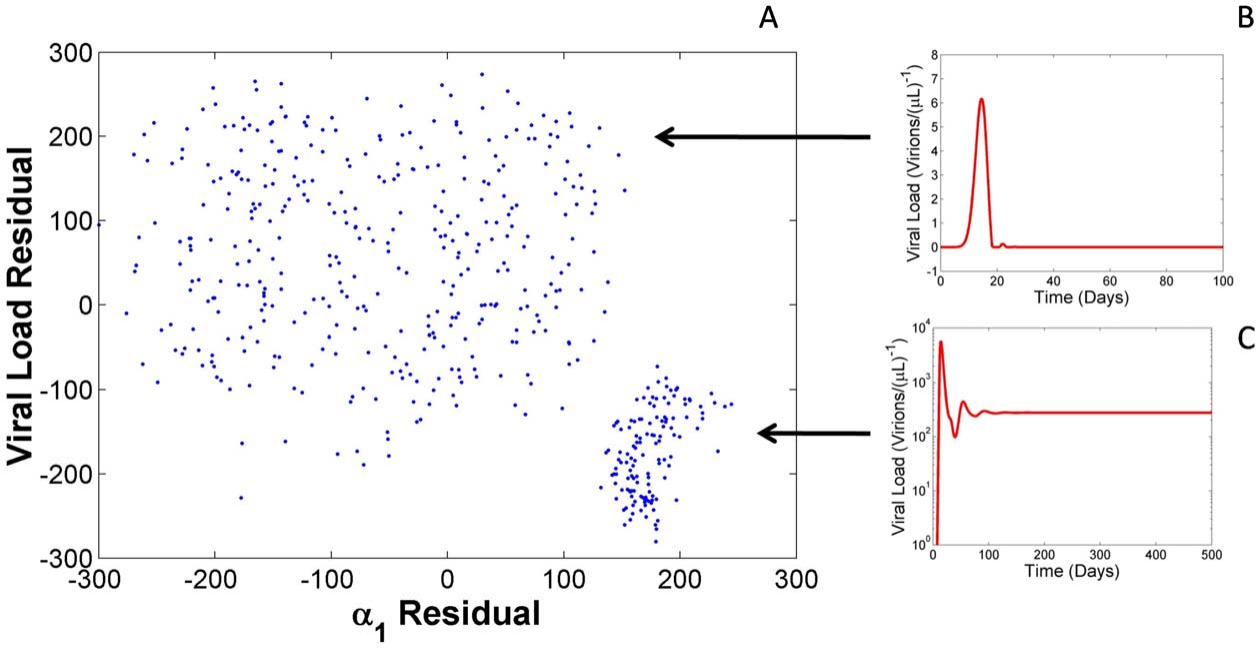
PRCC analysis of the 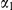 parameter reveals strong bifurcation. (A) A plot of the weighted PRCC residuals of 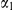 against viral load reveals two clusters. (B) Representative plot of cluster 1 viral dynamics. Parameter sets within cluster 1 were simulated and complete viral load clearance was seen within the first two weeks of infection for all points found within the cluster, thus signifying a largely non-biological dynamic. (C) Representative plot of cluster 2 viral dynamics. Parameter sets within cluster 2 revealed high steady state viral loads representative of the patient fits. Viral steady state was measured at t  =  200 days over a range of 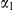 values from 0.0 to 1.0 (day)^1^.

Partial rank correlation coefficient (PRCC) analysis was performed in order to examine the importance of the individual parameters on viral load. PRCC isolates a single parameter for analysis independent of others (further explained in the methods) and examines its behavior in the system. This analysis is important in systems where parameters are highly sensitive or not well known in the literature, as individual contributions must be taken into account when making predictions. Our model considers well-defined parameters for viral infection as well as newly introduced parameters such as the rate at which normal Tregs downregulate CD4^+^ cells 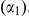 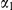 accounts for the rate of change of CD4+ cells in response to normal regulator T-cells. Previous models have not considered this CD4+ cell response directly but recent research has suggested regulatory T-cells do effect CD4+ cells throughout the infection process [3] [5]. We noticed a clear bifurcation in viral load behavior when isolating 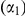 (Fig. 2A). Two clusters were observed (cluster 1 and 2) with divergent viral behaviors. Parameter sets within cluster 1 were simulated and complete viral load clearance was seen within the first two weeks of infection for all points found within the cluster, thus signifying a largely non-biological dynamic (Fig. 2B). Parameter sets within cluster 2 revealed high steady state viral loads representative of the patient fits (Fig. 2C).

### Sensitivity of Treg Downregulation of CD4^+^ on Viral Load

**Figure 3:**
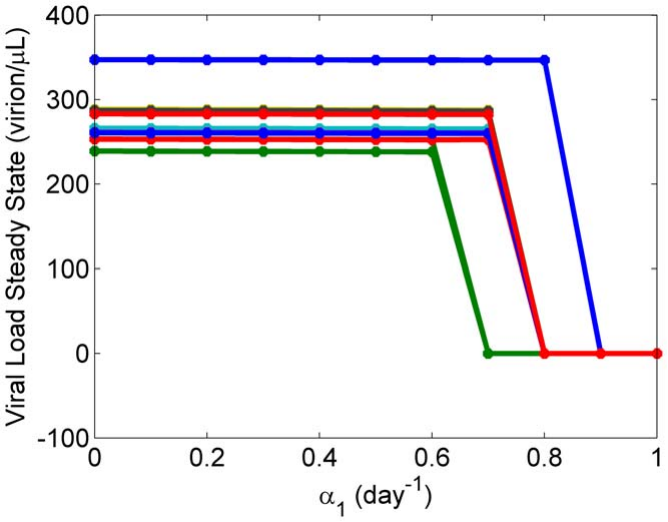
Sensitivity analysis of the 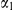 parameter. Representative simulations were chosen over a range of physiological parameter sets. Each data point shows the viral steady state, measured at 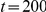 days, over a range of 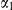 values from 0.0 to 1.0 (day)^−1^. Dynamics remain relatively unchanged over low values of 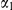 but drop significantly with sufficiently large 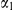 (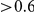 day^−1^) to the extent that there is viral clearance.

Bifurcated clustering was further examined through numerically sampling, physiologically bound parameter sets defined in Table 1 (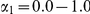
(day)^−1^). Since 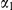 has values that are currently unknown in the literature, we first varied it over three orders of magnitude. All of our results, however, suggest that 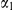 is bounded between 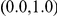. Viral loads at steady state (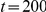 days) were recorded for each parameter set over the 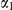 range. Simulations reveal 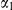 parameters to be (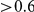) and (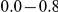) for clusters 1 and 2, respectively (Fig. 3A). Steady state behavior is relatively constant among the various curves; specifically, viral load seems unchanged over small values of 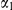, however, a clear bifurcation to zero occurs when 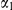 is sufficiently large (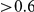 day^−1^). These results suggest a physiological range in normal Treg behavior.

### Sensitivity of Transition Rate of normal Tregs on Viral Load

**Figure 4:**
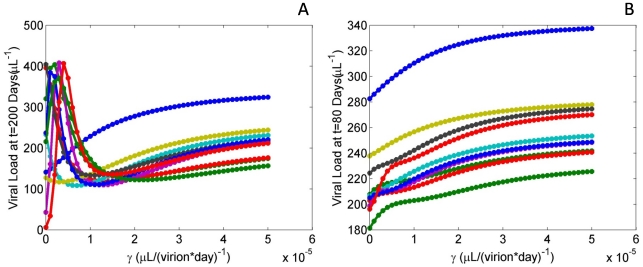
Sensitivity analysis of the 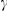 parameter. Graphs depicting 10 representative parameter sets, plotting viral load at various time points over varied values for 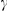. Each distinct curve represents simulations obtained from a single, randomly determined, physiological parameter set. (A) Plots for viral load measured at t = 80 days. For the majority of curves, viral load spikes at smaller values of 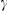 and then decreases to a steady state for higher 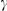 values. Several simulations reveal viral loads quite close to and at 0 virions/(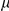
L)^−1^ for 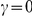. (B) Plots for viral load measured at t = 200 days. Most curves are seen to monotonically increase over increasing values of 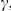 suggesting a direct relationship between high adaptive Treg production and high steady state viral loads.

Ranging 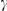 (the transition rate of normal Tregs into adaptive Tregs) revealed surprising dynamics in viral load, particularly during early infection (Fig. 4). It was seen that viral load was highly dependent on 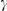 in the acute stage of infection (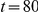 days), but was to a lesser degree dependent during steady state (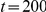 days). For some parameter sets, it was even found that 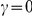 would result in viral clearance; thus for these simulations it was necessary to have an adaptive population for simulation to achieve steady state behavior. In addition, it was revealed that a larger value for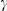, *i.e.*, a larger rate of nTregs becoming aTregs, results in, on average, a 20% increased viral load at steady state.

### Impact of Treg Parameters on Viral Load and Cellular Populations

**Figure 5:**
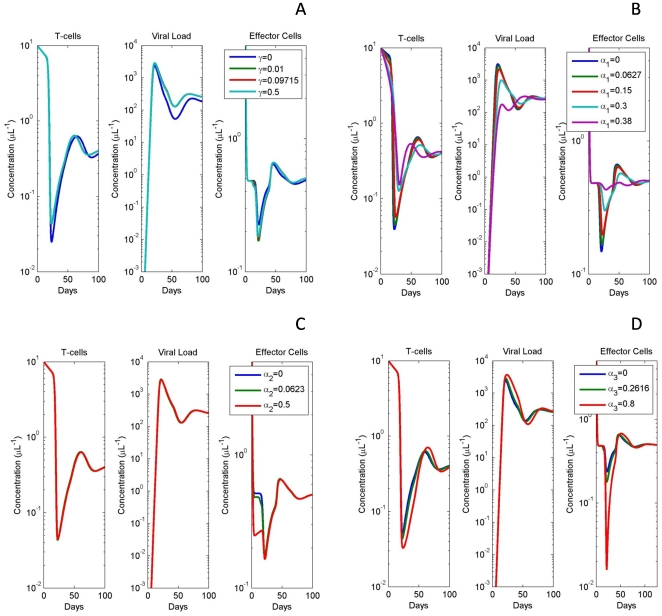
Plots of T-cell, Virus, and Effector populations with changes in regulatory T-cell parameters. Each distinct curve represents simulations from a unique randomly obtained, physiological parameter set. This figure depicts changes in T-cells, viral load, and effector cells given changes in regulatory T-cell parameters 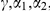 and 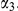 (A) 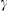 was varied and plotted alongside best fit of 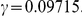 It was seen that there were two very distinct behaviors; one behavior for 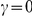 and one for 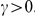 For 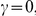 viral load could be seen to stay lower and effector cells remained slightly elevated while T-cells remained slightly lowered. (B) 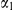 was varied and plotted alongside the best fit for 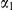 from data fitting which was 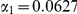 (days)^−1^. All changes occur on the acute stages of infection and it is seen that for large values of 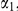 viral peak is significantly reduced. (C) 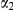 was varied and plotted alongside the best fit of 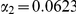 (days)^−1^. T-cell and viral load plots appear similar although it is seen that large values of 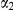 result in a sharp decrease in effector population during acute infection. (D) 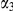 was varied and plotted alongside best fit of 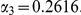 It was seen that for large values of 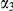 that effector population would drop drastically during the acute stage of infection and would result in a minor increase in viral load as well.

To elucidate the dynamics of the system, different populations were examined over increasing values of the Treg-related parameters 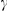 and 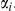 The best fit for Patient 8 was utilized as the starting parameter set, and the respective parameters were varied over several orders of magnitude (Fig. 5). T-cell concentration, viral load, as well as effector cell concentration (measured as the sum of immature and mature effectors) were graphed against 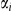 and 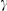 (Fig. 5). Through the plots, it was seen that steady state concentrations of the three populations were relatively unchanged. The exception was in the case of adaptive transition rate 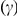 where low values of 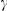 resulted in a moderate decrease in steady state viral load. However, it was generally seen that the main differences in dynamics occurred in the acute stage of infection, *i.e.* for 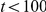 days.

An important finding was that 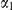 was found to be inversely proportional to peak viral load, suggesting that reducing the target population can limit the proliferation of virus during the early stages of infection. 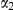 did not seem to have much of an effect on T-cell and virus dynamics; however for large values of 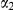, there was an early effector population drop which then rebounded to a higher level. When 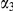 was increased, it was found that viral load would increase, as expected due to the fact that 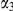 controls the rate of downregulation of mature effectors by adaptive Tregs. However, it was also found that large 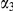 was inversely proportional to T-cells, which gave a surprising result (Fig. 5D). It can be seen that viral load can remain elevated during T-cell depletion (Fig. 5D). It was additionally found that 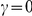 resulted in low viral load while 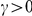 on the same order as the data fitting would result in an elevated viral load, suggesting adaptive Tregs can have a deleterious effect on the patient (Fig. 5A).

## Discussion

Although the behavior of regulatory T-cells in HIV infection is largely unknown, recent literature contrasts heavily on whether such T-cell populations are helpful or deleterious to the patient [3]. It has been theorized that there are two subsets of Tregs, a basal population of normal Tregs, and a population of adaptive Tregs derived from interactions between normal populations and viral particles [3] [6]. While the explicit mechanism for how such a population can emerge is still unknown, it has been hypothesized to occur at the transcriptional level resulting in differential regulatory T-cell distribution in various regions of the body [6]. Here, a physiologically relevant model of normal and adaptive Tregs was utilized to model patient viral loads and examine the effect of normal Treg parameter boundaries and an adaptive Treg subgroup.

Normal regulatory T-cells, specifically the efficiency of their clearance of CD4^+^ cells, were characterized throughout the model by the parameter 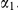 Using the analysis as discussed, 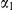 was observed to range from 0.0–0.7 day^−1^ due to a bifurcation in viral steady state at higher efficiencies. This suggests a defined range on normal Treg action and acts as a first step in studying the dynamics of this regulatory population within the framework of HIV infection. These bounds additionally suggest that nTreg efficiency in down-regulating CD4^+^ cells can drastically affect viral load behavior. Specifically, if efficiency is relatively high, the number of target cells for HIV is reduced enough to prevent proliferation of virus due to a clearance of the target cell population. The limits to which nTreg dynamics can be manipulated *in vivo* will elucidate the dynamics of regulatory T-cells within HIV dynamics and give light to potential therapeutic techniques against the infection.

Adaptive Tregs are suggested in our model to counteract the immune system’s defenses against HIV through depletion of the effector cell populations. We reproduce normal viral load behavior in the presence of aTregs (Fig. 1) and through the sensitivity dynamics of the aTreg parameter 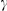 observe that, in some instances, the presence of an adaptive population is essential to produce realistic viral behavior. Additionally, the adaptive transition parameter, 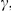 appears to influence viral loads; specifically, larger 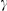 values result in higher steady state viral loads and increased T-cell counts (Fig. 5). Distinct biological markers will be necessary for further study of adaptive T-cell populations; however, the possibility of two classes of regulatory T-cells is plausible within the context of mature effector cell depletion. Additional mechanisms of aTreg action, such as depletion of other immune cell populations or other events would be of great interest to further examine Treg-HIV dynamics.

The use of two effector cell populations, immature and mature, proved successful in maintaining realistic cell population concentrations and while producing comparable viral fits to previous models. Sensitivity analyses reveal that varying parameters related to regulatory T-cells largely influence the acute stages of infection but are relatively robust at long times (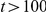 days). Also, we observe regulatory T-cells to be pivotal in early viral peaks and early depletion of CD4^+^ T-cells.

While production and death rates for the regulatory T-cells could be extrapolated from similar parameter values approximated in previous research, little was known about 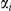 and 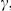 which reflect the downregulatory capabilities of the nTregs and aTregs as well as the rate at which normal Tregs migrate into the adaptive population. Parameter fitting gives bounds on these values and desirable visualization to differentials in viral load dynamics. Through further studies of Tregs and their different classes, new therapeutic techniques can be developed to potentially limit normal Treg action and reflect optimal viral load reduction providing additional insights into viral and immune system’s capabilities during infection.

## Methods

### Ethics Statement

There was no need for consent in the work presented here as no work with patients was performed.

To examine the dynamics of HIV viral load during primary infection, we propose a system of delay differential equations consisting of seven variables: normal Tregs (*R*), adaptive Tregs (*R_a_*), target CD4^+^ cells (*T*), productively infected CD4^+^ T cells (T^*^), free virus (*V*), immature CD8^+^ cells (*E_i_*) and mature CD8^+^ cells (*E_m_*).

### Normal and Adaptive Regulatory T-Cells

Here, we introduce normal Tregs as well as the hypothesized adaptive Tregs. It has been suggested that HIV interacts with normal Tregs and induces them into a secondary adaptive population which can have deleterious impacts on the patient and assist the virus. To model this dynamic, normal Tregs 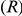 were given a production rate of 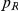 and a death rate of 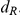 The death rate of adaptive Tregs 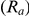 was defined by 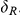 The mass-action term 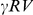 was utilized, in which 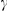 is the proportion of interactions between *R* and *V* which result in adaptive Tregs.

The following are the equations for the *R* and 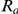 populations,
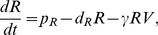
(1)

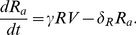
(2)


### CD4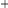 T-Cells and HIV Virus Load

For viral load, we use the same form as seen in the Perelson, Nelson 1999 model,
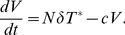
(3)


For the CD4^+^ population, an almost identical form as the Perelson, Nelson 1999 model is utilized, with a proliferation term *s*, a half-life of 1/*d*, and a bikinetic expression *kVT* to express the notion that interaction of virus with T-cells at some probability *k* results in the infection of T-cells. In the literature, it is well characterized that Tregs the proliferation rate of T-cells [1] [3]–[4]. Thus, the term 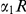 was introduced, which serves as the basal downregulation of CD4^+^ cells by the normal population of Tregs. The uninfected T-cell population equation is as follows,
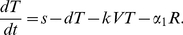
(4)


Infected T-cells were produced from the bikinetic interaction (*kVT*) in which uninfected T-cells interact with HIV to become infected. The half-life for these infected cells is 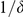 with a clearance term 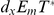; thus infected cells are cleared also through a bikinetic interaction with mature CD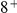 effectors at a rate 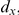


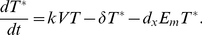
(5)


### Effector Cell Equations

Various models of HIV include or exclude cytotoxic CD8^+^ T-cells; depending on the specific dynamics of interest [9] [11]. In the laboratory, it has been observed that Tregs impact the activity of these cells, thus we include them as a key element in our model. Some previous models [9], use a single equation to represent effector cells, however, this population would often trend to non-physiological conditions. To adjust for this issue, we separated the effector population into two subsets: an immature class of effectors and a mature class. Since effector cells need to be presented with HIV-antigen in order to specifically combat infected cells, we write our equations with immature effectors interacting with HIV in order to become mature effectors.

For immature effectors, we assume similar dynamics to the uninfected T-cell equation. There is a constant rate of production 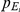, a death rate 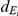 and a loss of cells due to conversion into mature effector cells, dependent on virus interaction at some rate 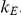 Additionally, there is a downregulation of the immature population by normal Tregs, expressed through the 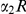 term,
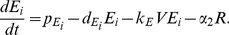
(6)


Mature effectors are produced through viral interaction with the immature effector population (*kVE_i_*) and die at a rate 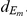 A time delay was used in the production term of the mature effectors to represent the biological lag that occurs between immature effector recognition of virus and the production of mature effectors. Adaptive Tregs are hypothesized to specifically downregulate the mature effector response through the expression 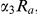 thus suppressing normal immune responses,
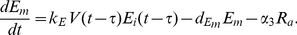
(7)


### Parameter Fitting

Viral load patient data were used from data collected in [12]. Fitting was accomplished through running 2,000 iterations using a Simplex optimization algorithm through JSim Software [13]. All parameters were varied with known parameters from [9] used as start values in the optimization; parameter descriptions, ranges and best fits can be found in Table 1. Parameters which were relatively unknown, for instance production rate of nTregs, were extrapolated from similar parameters and were varied over an order of magnitude to ensure that their starting points were biologically feasible. A bootstrapping algorithm was used in order to find confidence intervals for the parameters related to Treg dynamics (Table 1).

### PRCC Analysis and other Sensitivity Analysis

In the analysis of our model, we were interested in determining which parameters have a strong effect on key outputs such as viral load. This is especially important for parameters which are not well-characterized in the literature, which included terms related to normal and adaptive regulatory T-cell function; if these have a strong influence on model outputs, we must be careful to qualify any conclusions drawn from model analysis with the disclaimer that such conclusions may be very sensitive to rough parameter estimates. The results of a sensitivity analysis can be used as a guide in determining which parameters are most important, whether to re-express the model in terms of better-understood parameters, and potentially which parameters can be measured or investigated more thoroughly.

The Partial-Rank Correlation Coefficient (PRCC) measures the association between two variables, with the effects of other variables removed. Given a model parameter of interest and a model output at a particular time point, the PRCC can be used as a sensitivity metric of the output to the parameter, independent of the other parameters, as described in Marino et al. [14].

The first step of computing the PRCC of parameter 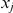 and output *y* is to rank-transform the data in variables 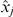 and 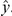 The PRCC is then given by the correlation between the residuals 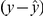 and 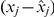, where, where 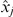 and 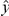 are given by [14],
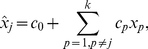
(8)

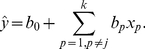
(9)


A high correlation between these residuals suggests that output 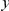 is sensitive to parameter 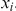 We can apply this technique to our HIV model by setting the model output to, for example, viral load at time 80 days after primary infection. PRCC analysis can give us a snapshot of the influential parameters on viral load at this time point.

In addition to PRCC analysis, a second sensitivity analysis was performed for the parameters 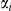 and 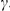 One of the parameters was isolated, and the other parameters were chosen through a Latin Hypercube Sampling (LHS). The isolated parameter was ranged over several orders of magnitude and for each value of the parameter, viral load was simulated and recorded at 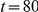 days as well as 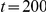 days. Plots of viral load vs. parameter value were constructed to depict the dynamics. Analysis was performed for 100 distinct parameter sets, with representative plots in (Fig. 3, 4). In both cases, we find using the PRCC analysis that 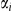 and 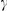 are robust parameters and viral dynamics are not highly sensitive to small perturbations in either parameter, suggesting that our new model is more robust then previous models.
